# Parkinson’s Disease Medication Alters Small Intestinal Motility and Microbiota Composition in Healthy Rats

**DOI:** 10.1128/msystems.01191-21

**Published:** 2022-01-25

**Authors:** Sebastiaan P. van Kessel, Amber Bullock, Gertjan van Dijk, Sahar El Aidy

**Affiliations:** a Host-Microbe Interactions, Groningen Biomolecular Sciences and Biotechnology Institute, University of Groningen, Groningen, The Netherlands; b Department of Behavioral Neuroscience, Groningen Institute for Evolutionary Life Sciences, University of Groningen, Groningen, The Netherlands; University of California, San Francisco

**Keywords:** Parkinson’s disease treatment, pramipexole, ropinirole, levodopa, dopamine, small intestinal bacterial overgrowth, gut motility, microbiota, drug side effects

## Abstract

Parkinson’s disease (PD) is known to be associated with altered gastrointestinal function and microbiota composition. To date, the effect of PD medication on the gastrointestinal function and microbiota, at the site of drug absorption, the small intestine, has not been studied, although it may represent an important confounder in reported microbiota alterations observed in PD patients. To this end, healthy (non-PD) wild-type Groningen rats were employed and treated with dopamine, pramipexole (in combination with levodopa-carbidopa), or ropinirole (in combination with levodopa-carbidopa) for 14 sequential days. Rats treated with dopamine agonists showed a significant reduction in small intestinal motility and an increase in bacterial overgrowth in the distal small intestine. Notably, significant alterations in microbial taxa were observed between the treated and vehicle groups; analogous to the changes previously reported in human PD versus healthy control microbiota studies. These microbial changes included an increase in *Lactobacillus* and *Bifidobacterium* and a decrease in *Lachnospiraceae* and *Prevotellaceae*. Markedly, certain *Lactobacillus* species correlated negatively with levodopa levels in the systemic circulation, potentially affecting the bioavailability of levodopa. Overall, the study highlights a significant effect of PD medication intrinsically on disease-associated comorbidities, including gastrointestinal dysfunction and small intestinal bacterial overgrowth, as well as the gut microbiota composition. The results urge future studies to take into account the influence of PD medication *per se* when seeking to identify microbiota-related biomarkers for PD.

**IMPORTANCE** Parkinson’s disease (PD) is the second most common neurodegenerative disorder and is known to be associated with altered gastrointestinal function and microbiota composition. We previously showed that the gut bacteria harboring tyrosine decarboxylase enzymes interfere with levodopa, the main treatment for PD (S. P. van Kessel, A. K. Frye, A. O. El-Gendy, M. Castejon, A. Keshavarzian, G. van Dijk, and S. El Aidy, Nat Commun 10:310, 2019). Although PD medication could be an important confounder in the reported alterations, its effect, apart from the disease itself, on the microbiota composition or the gastrointestinal function at the site of drug absorption, the small intestine, has not been studied. The findings presented here show a significant impact of commonly prescribed PD medication on the small intestinal motility, small intestinal bacterial overgrowth, and microbiota composition, irrespective of the PD. Remarkably, we observed negative associations between bacterial species harboring tyrosine decarboxylase activity and levodopa levels in the systemic circulation, potentially affecting the bioavailability of levodopa. Overall, this study shows that PD medication is an important factor in determining gastrointestinal motility and, in turn, microbiota composition and may, partly, explain the differential abundant taxa previously reported in the cross-sectional PD microbiota human studies. The results urge future studies to take into account the influence of PD medication on gut motility and microbiota composition when seeking to identify microbiota-related biomarkers for PD.

## INTRODUCTION

The microbiota composition of patients with Parkinson’s disease (PD) has been compared to that of healthy controls (HC) in an extensive amount of studies (mainly cross-sectional) (1–16). Nevertheless, there is low consensus among the findings in these reports, making it nearly impossible to determine whether the changes in microbiota composition are causally linked to the disease or due to confounding factors such as PD medication, which tends to vary between individuals (17). Indeed, PD medication is a major differentiating factor between PD patients and HC subjects and only a few studies (1, 3, 6, 8, 14) investigated or reported the effect of medication on the microbiota profiles of fecal samples from PD patients.

The large surface area of the small intestine results in complete absorption and delivery of the majority of ingested drugs, including PD medication, occurring at that site of the intestine (18, 19). In fact, Maier et al. estimated that 17% of the investigated drugs is excreted in feces (20), implying that the remaining 83% is likely to be absorbed in the small intestine and plausibly affect the residing microbiota *in situ*.

However, to date, no studies have been performed in HC subjects to determine whether the PD medication intrinsically affects the microbiota composition, irrespective of the disease itself, at the site of drug absorption, the small intestine.

Most PD medications work through their effect on the dopaminergic system in the brain. Additionally, PD medication exerts influence on the peripheral dopaminergic pathways in the enteric nervous system (ENS) (21, 22) and the immune system (23, 24). Dopamine and/or dopamine agonists are known to affect gut motility in rodents, dogs, and humans (25–39). Gut motility is usually inferred by Bristol stool score, which is known to be a major contributor to the variation in the fecal microbiota composition (40). Many PD patients experience nonmotor symptoms, including gastrointestinal (GI) dysfunction, which is typically displayed as reduced small intestinal motility (41, 42). Notably, decreased small intestinal motility is one of the causes of small intestinal bacterial overgrowth (SIBO) (43)—a condition that is significantly more prevalent in PD patients (up to 54.5%) (44–46).

Recently, PD medication has also been associated with the development of GI symptoms (47) or slow GI transit (48) in PD patients. Analogously, we showed that the unabsorbed residues of levodopa that reach the distal small intestine is converted to a bioactive molecule, which reduces ileal contractility in mice *ex vivo* (49). Nonetheless, whether PD medication *per se* is also associated with alterations in microbiota composition, small intestinal gut motility, and SIBO remains unknown.

In this study, using healthy rats, we showed that pramipexole and ropinirole, two commonly prescribed PD medications, have profound effects on the gut microbiota and small intestinal motility, irrespective of any PD symptoms.

## RESULTS

### Parkinson’s disease medication reduces small intestinal motility in wild-type Groningen rats.

**FIG 1:**
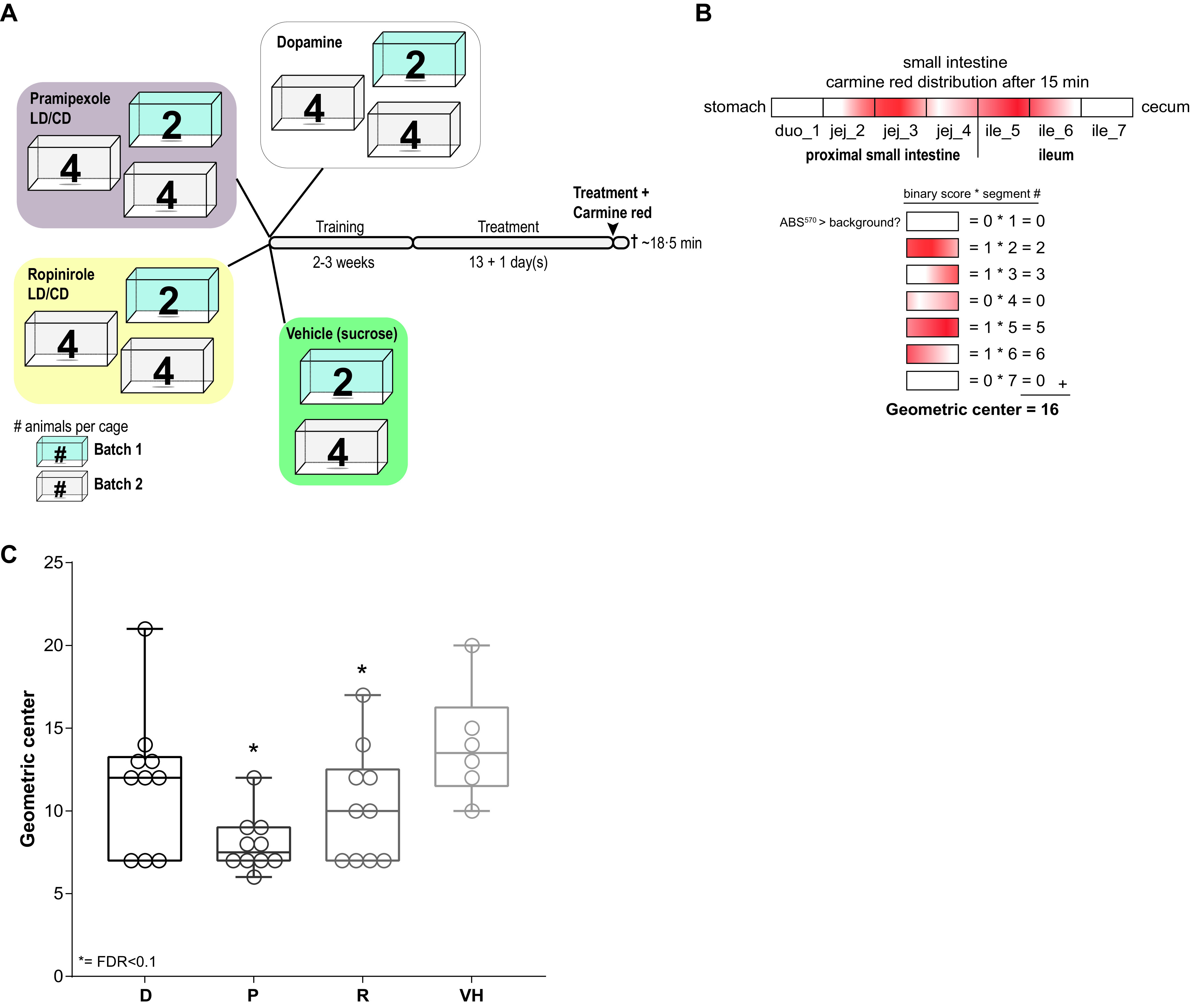
Small intestinal motility is affected by PD medication treatment. (A) Setup of the animal experiments. The experiment was performed with two independent batches of 2 to 4 animals per cage. After training with 10% sucrose (2 to 3 weeks), animals were treated with PD medication for 14 days. On the last treatment day, all animals received their treatment with the addition of 1.2% (wt/vol) carmine red (the vehicle and dopamine groups received levodopa-carbidopa with carmine red). All animals were sacrificed after on average at 18.5 min. (B) Schematic representation of the small intestine. Each rectangle represents a different section assessed, where carmine red distribution in the small intestine is depicted in red. Each segment was scored in a binary fashion and multiplied by the segment number, resulting in the geometric center. duo, duodenum; jej, jejunum; ile, ileum. (C) Geometric center per treated group. D, dopamine; P, pramipexole; R, ropinirole; VH, vehicle (10% sucrose). Boxes represent medians with interquartile ranges, and whiskers represent the maxima and minima. Significance compared to VH (asterisks) was tested with one-way ANOVA followed by Fisher’s LSD test with FDR correction.

To test whether commonly prescribed PD medication affects the motility in the small intestine, the main site of PD drug absorption (50), wild-type Groningen (WTG) rats were employed and were treated for 14 sequential days with dopamine (D), pramipexole (P, in combination with levodopa-carbidopa), ropinirole (R, in combination with levodopa-carbidopa), or vehicle (VH) (Fig. 1A). Pramipexole and ropinirole were combined with levodopa-carbidopa, as these medications are often coprescribed in PD treatment (51). Although dopamine is not used as a treatment for PD, it was included in the study for two reasons: (i) it acted as a control for the dopamine agonist groups, and (ii) PD patients usually have a higher exposure (2.5- to 40-fold) to dopamine—a metabolite of the levodopa treatment—than HC subjects (52–54). On the last treatment day, animals were sacrificed 18.5 ± 0.68 min after the administration of the PD medication in combination with carmine red. No significant differences (one-way analysis of variance [ANOVA]: *F* = 0.4977, *P* = 0.6865) at the time of sacrifice (i.e., time after last treatment) were observed between the studied groups (see Fig. S1A in the supplemental material). The small intestine was sectioned into a total of 7 parts, and their contents were assessed for carmine red spectrophotometrically. Carmine red detection was scored in a binary fashion per segment (detection was scored as 1; no detection was scored as 0), and the geometric center, a sensitive and reliable measure of intestinal transit (55), was determined (Fig. 1B). Pramipexole- and ropinirole-treated groups showed significant mean decreases of −1.8-fold and −1.4-fold in the geometric center, respectively, resulting in an ∼30% reduced small intestinal motility compared to the vehicle-treated group (Fig. 1C). In contrast, the dopamine-treated group did not show a significant effect (−1.2-fold mean decrease) on the small intestinal motility, although 8/10 (up to the 3rd quartile) of the points were below the median for the vehicle group. These findings suggest that PD medication intrinsically affects small intestinal motility, which may, in turn, influence the bacterial composition, potentially increasing the risk of developing SIBO at the site of drug absorption, namely, the small intestine.

10.1128/mSystems.01191-21.1 FIG S1 No difference in time of sacrifice after final carmine red treatment. Box plots represent the median time of the blood sample (i.e., time after last treatment) with interquartile range, and whiskers represent the maxima and minima. Significance was tested using a one-way ANOVA. Download FIG S1, EPS file, 1.9 MB. Copyright © 2022 van Kessel et al. 2022 van Kessel et al. https://creativecommons.org/licenses/by/4.0/ This content is distributed under the terms of the Creative Commons Attribution 4.0 International license.

SIBO is defined as an overgrowth of more than 10^5^ CFU/mL in the human proximal bowel (43). In rats, it was shown that the migrating motor complex (MMC) correlated strongly with the bacterial counts in the small intestine (56). Therefore, we quantitatively determined the CFU in the proximal small intestinal (duodenum and jejunum) and ileal content (Table 1). In the proximal small intestine, no significant differences were observed between the treated and vehicle groups (Table 1). In contrast, there was a significant increase in bacterial counts in the ileal content of the treated groups compared to the vehicle (Table 1). Only the ropinirole-treated animals had significantly higher bacterial counts on both the aerobically (2.7-fold mean increase; *P* = 0.024, *q* = 0.073) and anaerobically (3.6-fold mean increase; *P* = 0.020, *q* = 0.061) incubated plates, while the pramipexole-treated animals showed a borderline nonsignificant increase in the anaerobic (3.1-fold mean increase; *P* = 0.054, *q* = 0.080) and strictly anaerobic (6.8-fold mean increase; *P* = 0.046, *q* = 0.138) counts (Table 1). Collectively, the results imply that the reduction in gut motility caused by PD medication is plausibly associated with the observed increase in bacterial counts in the ileum of the treated groups.

**TABLE 1:** Bacterial counts from rat small intestine^*a*^

Agent	Median CFU/mL (IQR)					
	Aerobic		Anaerobic		Strictly anaerobic	
	Proximal small intestine	Ileum	Proximal small intestine	Ileum	Proximal small intestine	Ileum
D	1.7E+7 (3.1E+6 to 1.5E+6)	3.6E+7 (2.5E+7 to 4.2E+6)	4.2E+7 (1.6E+7 to 2.3E+6)	5.9E+7 (4.1E+7 to 1.4E+7)	1.1E+7 (7.0E+6 to 8.0E+5)	2.2E+7 (1.0E+7 to 5.0E+6)
P	2.4E+7 (1.5E+7 to 2.2E+6)	5.6E+7 (2.5E+7 to 1.4E+7)	6.2E+7 (3.8E+7 to 4.6E+6)	3.4E+8 (6.8E+7 to 2.1E+7)#	2.6E+7 (2.1E+7 to 2.4E+6)	1.2E+8 (4.0E+7 to 2.7E+6)##
R	1.0E+7 (4.6E+6 to 1.3E+6)	1.8E+8 (7.5E+7 to 3.6E+7)*	2.7E+7 (1.0E+7 to 3.9E+6)	2.8E+8 (1.2E+8 to 4.4E+7)*	1.3E+7 (2.6E+6 to 7.0E+5)	1.7E+8 (1.9E+7 to −1.8E+8)
VH	3.5E+7 (9.5E+6 to 2.6E+6)	2.4E+7 (2.1E+7 to 7.0E+6)	6.9E+7 (2.8E+7 to 3.8E+6)	7.0E+7 (4.8E+7 to 3.4E+7)	3.5E+7 (1.7E+7 to 1.2E+6)	2.5E+7 (1.1E+7 to −3.0E+7)

a The proximal small intestinal and ileal CFU/mL were counted after 48 h aerobic or anaerobic incubation of jejunal and ileal content. Strict anaerobic counts were estimated by subtracting the anaerobic counts with the aerobic counts. IQR, interquartile range; D, dopamine; P, pramipexole; R, ropinirole; VH, vehicle (10% sucrose). Extreme outliers were removed using the ROUT method (*Q* = 0.1%). Significance, compared to VH, was tested with one-way ANOVA followed by Fisher’s LSD test with FDR correction (*q* < 0.1). #, *P* = 0.054, *q* = 0.080; ##, *P* = 0.046, *q* = 0.138.

### Parkinson’s disease medication alters the microbiota composition.

**FIG 2:**
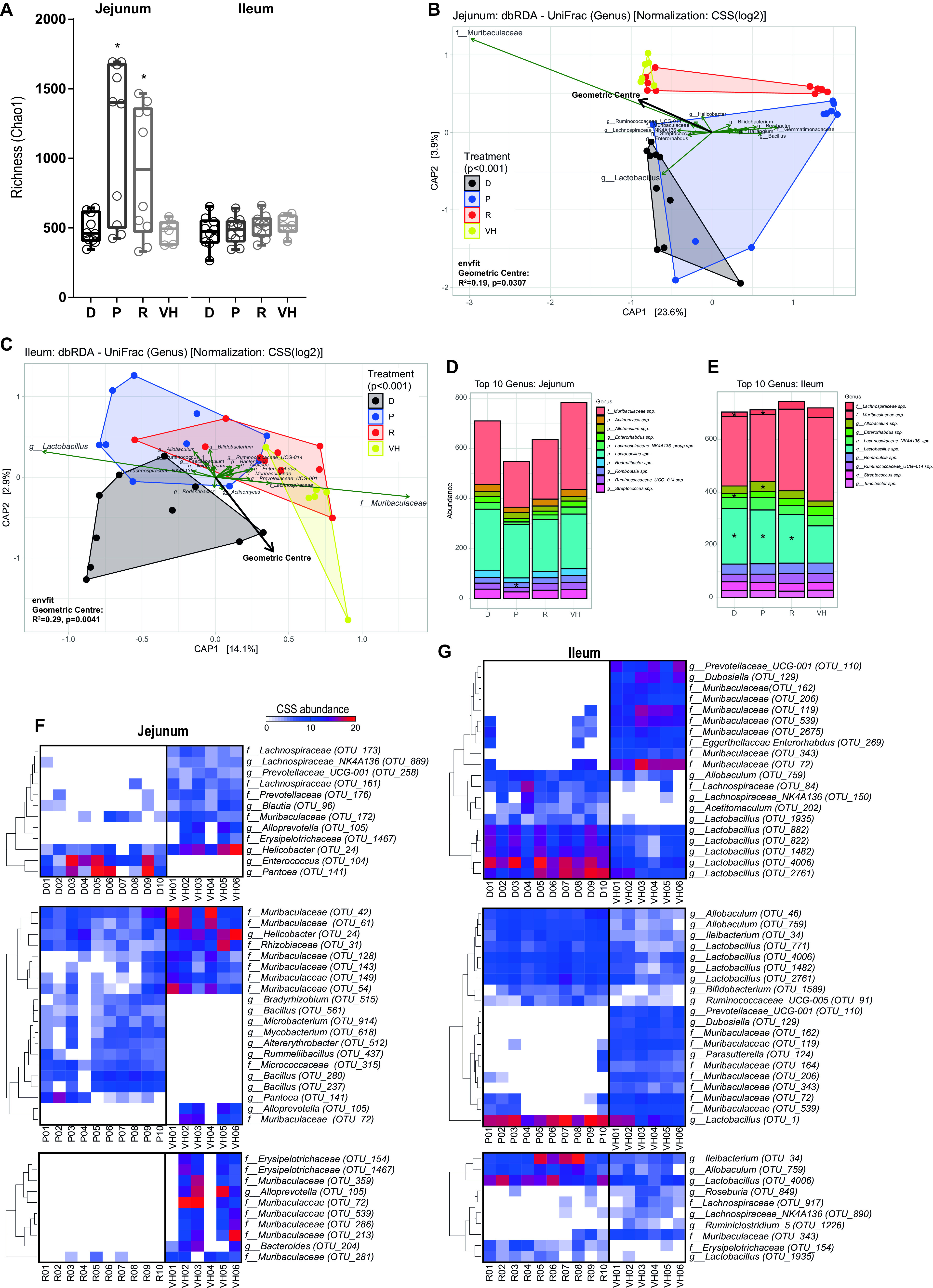
PD medication and geometric center contribute significantly to the variation in the microbiota composition. (A) Species richness (Chao1) of the proximal small intestine and ileum. Boxes represent medians with interquartile ranges, and whiskers represent the maxima and minima. Significance compared to VH (asterisks) was tested with one-way ANOVA followed by Fisher’s LSD test with FDR correction. (B and C) dbRDA using unweighted UniFrac distances at the genus level using CSS-scaled data of the proximal small intestine (B) and ileum (C) constrained for treatment. D, dopamine; P, pramipexole; R, ropinirole; VH, vehicle (10% sucrose). Significant contribution of the constrained variable to the variance of the dbRDA was tested with an ANOVA-like permutation test (anova.cca function in the R package vegan). Environmental vector (geometric center) fitting was performed using the envfit function in the R package vegan. (D and E) Stacked-bar plots with mean genus levels using CSS-scaled data from the top 10 taxa are from the proximal small intestine (D) and ileum (E). The asterisks indicate statistical significance compared to VH group tested using one-way ANOVA followed by Dunnett’s test. (G and H) Heat maps representing the extracted features for the LEfSe analysis (top 10) of the different treated groups of the proximal small intestine (G) and ileum (H). Significance was tested using one-way ANOVA followed by a Kruskal-Wallis (KW) test and LDA. A feature was considered significant when the KW *P* value was <0.01 and log(LDA score) was >2. For all the significant features, see Table S1. Heat map dendrograms represent Euclidian distance.

Next, we investigated whether the PD medication resulted in changes in the small intestinal microbiota composition directly or indirectly through altered small intestinal motility (Fig. 1B). To this end, we performed amplicon metagenomic sequencing on the V3 and V4 regions of the bacterial 16S genes. Interestingly, the richness (i.e., the number of different species observed) in the proximal small intestine, but not in the ileum, was significantly different in the pramipexole- and ropinirole-treated groups compared to the vehicle group (Fig. 2A). To determine whether the treatments affected the proximal small intestinal or ileal microbiota compositions, β-diversity analyses using distance-based redundancy analysis (dbRDA) with UniFrac distance constraining for treatment was performed. The analysis showed that the treatment had a significant effect on the microbiota composition in both proximal small intestine (Fig. 2B) and ileum (Fig. 2C) and that the samples were distanced further from the vehicle in the proximal small intestine than in the ileum. *Muribaculaceae* and *Lactobacillus* contributed most strongly to the observed variation (Fig. 2B and C) in both the proximal small intestine and ileum.

10.1128/mSystems.01191-21.3 TABLE S1 LEfSe of bacterial OTUs with significant differential abundance in samples treated with dopamine (D), pramipexol (P), ropinirole (R), and the vehicle (VH) collected from proximal intestine (PI) and ileum. Download Table S1, XLSX file, 0.02 MB. Copyright © 2022 van Kessel et al. 2022 van Kessel et al. https://creativecommons.org/licenses/by/4.0/ This content is distributed under the terms of the Creative Commons Attribution 4.0 International license.

Because the gut motility was significantly affected in the dopamine agonist-treated groups (Fig. 1B), the geometric center (i.e., the small intestinal transit time) was also tested for its association with the observed changes in the microbiota composition. Indeed, the geometric center significantly contributed to the altered microbiota composition caused by the different treatments in the proximal small intestine (*R*^2^ = 0.19, *P* = 0.0307) and ileum (*R*^2^ = 0.29, *P* = 0.0041). Overall, the results indicate that the PD medication and dopamine-agonist treatments affected the small intestinal bacterial composition, seemingly due to the treatment-associated alteration in gut motility.

To further identify which bacterial genera are most significantly altered by the treatment, differential abundance analysis was performed focusing on the 10 most abundant taxa in all groups. In the proximal small intestine, only *Romboutsia* spp. were significantly decreased (Dunnett’s test, *P* = 0.022) in the pramipexole-treated group compared to the vehicle (Fig. 2D). In the ileum, *Lachnospiraceae* spp. were decreased in both dopamine- and pramipexole-treated groups (Dunnett’s test, *P* = 0.033 and 0.034, respectively), while *Enterorhabdus* spp. were decreased in the dopamine-treated group and *Allobaculum* spp. increased in the pramipexole-treated group (Dunnett’s test, *P* = 0.011 and 0.002, respectively) compared to the vehicle group (Fig. 2E). Analogous to the dbRDA analysis (Fig. 2C), *Lactobacillus* spp. were significantly increased in the dopamine-, pramipexole-, and ropinirole-treated groups compared to the vehicle group (Dunnett’s test, *P* = 0.001, 0.003, and 0.047, respectively) (Fig. 2E).

Next, we used linear discriminant analysis (LDA) effect size (LEfSe) (57) for differential abundance analysis on the operational taxonomic unit (OTU) level (Table S1). Among the top 10 (per group) OTU hits, the main discriminant feature separating the vehicle from the other treatment groups was species from the family *Muribaculaceae* in both the proximal small intestine and ileum (Fig. 2F and G). Species belonging to the genus *Lactobacillus* were the main discriminant feature separating the groups treated with dopamine or PD medication from the vehicle group; this finding is in accordance with the results observed in Fig. 2B and C to E. Species from the families *Prevotellaceae*, *Lachnospiraceae*, and *Muribaculaceae* in the proximal small intestine or ileum were significantly decreased in almost all treated groups compared to the vehicle group, while species from the genus *Lactobacillus* were significantly increased in the ileum. Among the other significantly increased differential taxa were *Bifidobacterium* in the ilea of the pramipexole-treated group and *Enterococcus* in the proximal small intestines of the dopamine-treated group. Overall, these findings are highly relevant, since *Prevotellaceae* and *Lachnospiraceae* taxa are frequently reported to be decreased while *Bifidobacterium* and *Lactobacillus* taxa are increased in PD patients compared to HC subjects (17).

### *Lactobacillus* OTUs negatively associate with levodopa plasma availability.

**FIG 3:**
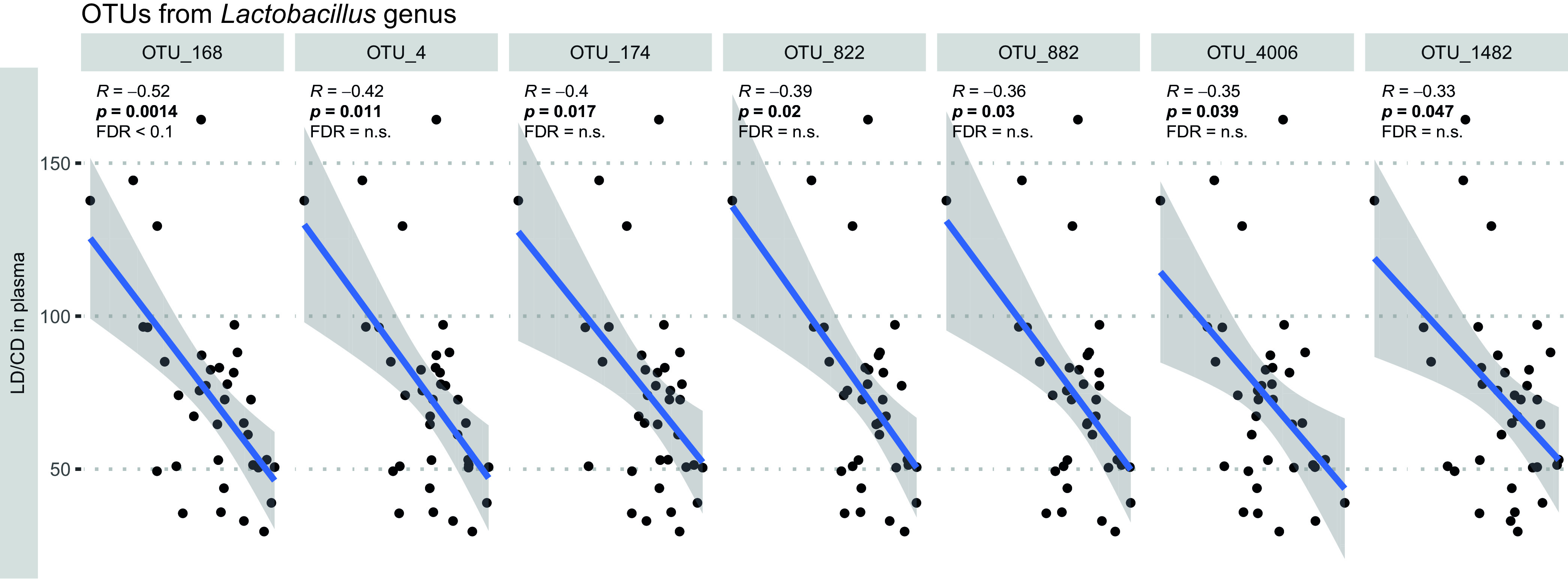
Lactobacillus OTUs are negatively associated with levodopa levels in the blood circulation. Graphs show linear models and Spearman correlations of the significant OTUs of the 50 most abundant OTUs with the levodopa uptake. Only *Lactobacillus* OTU_168 remained significant after FDR correction. For the nonsignificant OTUs, see Table S2. D, dopamine; P, pramipexole; R, ropinirole; VH, vehicle (10% sucrose).

In particular, the altered abundance of *Enterococcus* and *Lactobacillus*, which harbor a tyrosine decarboxylase enzyme, is crucial for possible gut bacterial interference with the availability of levodopa in the small intestine, as we previously showed (50). Subsequently, levodopa decarboxylase activity was measured in the proximal small intestinal and ileal samples, and the levodopa uptake was measured in blood samples. Around 75% of the proximal small intestinal and ileal samples showed levodopa decarboxylase activity. Remarkably, of the 50 most abundant OTUs in the proximal small intestine (1.2% of the total), there were significant negative correlations (Spearman correlations) only between the genus *Lactobacillus* (*Lactobacillus* sp. OTU_168 remained significant after false discovery rate [FDR] correction) and the plasma levels of levodopa-carbidopa when all treatment groups were combined (Fig. 3; also, see Table S2), while no significant differences were observed in levodopa uptake between the tested groups (Fig. S2A). Collectively, these results imply an interference of levodopa uptake by *Lactobacillus* species, as we previously reported (50).

10.1128/mSystems.01191-21.2 FIG S2 Levodopa uptake levels in the blood circulation per treatment group. The levodopa-carbidopa ratio in plasma at the maximum concentration (*C*_max_) is shown. D, dopamine; P, pramipexole; R; VH, vehicle (10% sucrose). Boxes represent medians with interquartile ranges, and whiskers represent the maxima and minima. Significance compared to VH (asterisks) was tested with one-way ANOVA followed by Fisher’s LSD test with FDR correction. Download FIG S2, EPS file, 1.9 MB. Copyright © 2022 van Kessel et al. 2022 van Kessel et al. https://creativecommons.org/licenses/by/4.0/ This content is distributed under the terms of the Creative Commons Attribution 4.0 International license.

10.1128/mSystems.01191-21.4 TABLE S2 Spearman correlation between the 50 most abundant OTUs in the proximal small intestine and the plasma levels of levodopa-carbidopa combining all treatment groups. Download Table S2, XLSX file, 0.01 MB. Copyright © 2022 van Kessel et al. 2022 van Kessel et al. https://creativecommons.org/licenses/by/4.0/ This content is distributed under the terms of the Creative Commons Attribution 4.0 International license.

## DISCUSSION

This study unraveled the effect of dopamine and the PD medications pramipexole and ropinirole in combination with levodopa-carbidopa on small intestinal motility and the associated alteration in the microbiota composition in healthy rats. Decreased small intestinal motility is a determining factor in the development of SIBO (43) and is prevalent in PD patients (44–46). Additionally, PD medication has been shown to be associated with GI symptoms (47) and increased transit times (48). Dopamine is not used as a PD treatment, as dopamine alone cannot pass the blood-brain barrier. However, dopamine can still be produced from levodopa endogenously by the human dopa decarboxylase (DDC) or exogenously via bacterial tyrosine decarboxylases (TDC) in the periphery (50). Despite the substantial number of reports describing an effect of dopamine on gut motility (26–39), dopamine did not exert a significant effect on the gut motility in our study (Fig. 1). This could be due to the metabolism of the drug during the absorption process. For example, in dogs, the first-pass metabolism of dopamine predominantly occurs in the small intestine, with an estimated bioavailability of only 3% and a half-life of 10.8 min (58). In contrast to dopamine, pramipexole and ropinirole, both of which showed a significant effect on the small intestinal motility (Fig. 1), have a much higher bioavailability with a longer half-life. Pramipexole has an oral bioavailability of ∼90%, a long half-life (between 11.6 and 14.1 h), and minimal metabolism (70 to 78% excreted unchanged in urine) and is primarily eliminated via the kidney (59). Ropinirole has an oral bioavailability of ∼50% and a shorter half-life of approximately 6 h (ranging from 2 to 10 h), and only 10% is excreted unchanged in urine and cleared by hepatic metabolism (60). The observation that 8/10 of the points (up to the 3rd quartile) in the dopamine group are below the median of the VH group (Fig. 1) still implies that dopamine could affect gut motility, but to a lesser extent than dopamine agonists.

Importantly, gut motility contributed significantly to the variation observed in the microbiota profiles, while the faster transit times (higher geometric center) associated closely with the vehicle group (Fig. 2B and C). Moreover, both dopamine and its agonists-treated groups shared similar distinct abundant taxa compared to the vehicle group (Fig. 2 and Table S1), implying that dopamine, pramipexole, and ropinirole act through similar mechanisms, likely by altering gut motility and consequently the microbiota composition.

Although the microbial profiles were altered in both the proximal small intestine and ileum, a clear separation was observed between the treated and untreated groups in the proximal small intestine compared to the ileum (Fig. 2). This increased alteration in the proximal small intestinal microbiota composition upon exposure to PD medication implies that the medication has a stronger effect on the proximal small intestinal motility. This effect could be due to the rapid absorption of the drugs in the proximal small intestine (58, 60, 61), resulting in the highest local drug concentration in the proximal small intestine. Plausibly, these drugs could also elicit a direct effect on the microbiota, which warrants further elucidation.

The increase in *Lactobacillus* and *Bifidobacterium* and the decrease in *Lachnospiraceae* and *Prevotellaceae* observed in our healthy rat model have been reported as a common finding among several studies investigating the fecal gut microbiota composition between PD patients and HC subjects (17). Although the comparison between rat small intestinal content and human feces should be made with caution, in rats, 85.9% of the taxa in the small intestine have been detected in feces (Spearman’s *R* = 0.69, *R*^2^ = 0.48, and *P* < 2.2E−16 on log-transformed data, calculated from Table S3 in reference 62) and are ultimately washed out through the large intestine. Additionally, analysis of microbiota composition in fecal samples from rats reported that they share higher levels of relative abundances with the human core fecal microbiota than those from mice (63). This suggests that the observed changes in microbiota composition in PD patients are, at least partly, due to the PD medication and not the disease *per se*. Intriguingly, *Lactobacillus* spp. were found to be the discriminating factor between all treated groups and the vehicle in the ileum (Fig. 2E and G). This is in agreement with the study by Romano et al. (17), where this genus was also the most strongly enriched in PD patients in the sequence data collected from various studies comparing the microbiota profile between PD patients and HC subjects. Remarkably, a significant negative correlation was observed between species from the genus *Lactobacillus* and levodopa uptake (Fig. 3). Several *Lactobacillus* species harbor the tyrosine decarboxylase enzymes, which can reduce levodopa (50). Thus, the observed association implies that the higher abundance of *Lactobacillus* species observed could have reduced the levodopa levels in the systemic circulation, potentially affecting the levodopa bioavailability.

Overall, this study shows the impact of commonly prescribed PD medications and dopamine on small intestinal motility, SIBO, and microbiota composition, irrespective of the PD. Importantly, the microbial alterations observed in our healthy rat model are consistent with the microbial alterations observed in human PD cross-sectional studies. Taken together, our findings highlight the urgency of taking PD medication into consideration when assessing alterations in the PD-associated microbiota.

## MATERIALS AND METHODS

### Rat experiments.

All animal procedures were approved by the Groningen University Committee of Animal experiments (approval number AVD1050020197786) and were performed in adherence to the NIH *Guide for the Care and Use of Laboratory Animals* (64).

Thirty-six adult male WTG rats (ages, 22 to 27 weeks) housed 2 to 4 animals/cage had *ad libitum* access to water and food (Altromin 1414) in a temperature (21 ± 1°C)- and humidity (∼60% relative humidity)-controlled room, with a 12-h light/dark cycle.

The rats were trained to drink 10% (wt/vol) sucrose solution from a burette with spout as follows. On 9 to 13 occasions over a period of 2 to 3 weeks, rats were taken from their social housing cage in the beginning (within 1 h) of the dark-phase cycle and placed in an individual training cage (length × width × height = 25 × 25 × 40 cm), without bedding, food, or water. Ten minutes after transfer to the training cages, rats were given a drinking burette with a 2.5-mL sucrose solution (10% [wt/vol]). On 2 to 4 training occasions, 1.2% carmine red (C1022; Sigma) was added to the sucrose solution. Over the course of training, all rats were trained to drink the sucrose solution avidly.

After 2 to 3 weeks, when the training was complete, animals were assigned at random to four different treatments groups, dopamine (D; *n* = 10), pramipexole-levodopa-carbidopa (P; *n* = 10), ropinirole-levodopa-carbidopa (R; *n* = 10), and vehicle (VH; *n* = 6). For each treatment group, the animals were in 2 or 3 different cages but were treated per cage to avoid cage bias as well as any effect of coprophagy. Rats in the designated groups were treated for 14 consecutive days as follows: (i) an average of 1.5 mg/kg dopamine (H8502; Sigma), (ii) an average of 0.0625 mg/kg pramipexole (A1237; Sigma) with 7.5/1.875 mg/kg levodopa-carbidopa (D9628/C1335; Sigma), (iii) an average of 0.15 mg/kg ropinirole (R2530; Sigma) with 7.5/1.875 mg/kg levodopa-carbidopa, or (iv) 10% sucrose (wt/vol) solution as vehicle only (VH). All treatments were dissolved in 2.5 mL 10% sucrose solution. Based on a person weighing 80 kg, the dosages are equivalent to 600/150 mg/day levodopa-carbidopa, 5 mg/day pramipexole, 12 mg/day ropinirole, or 120 mg/day dopamine based on 10% of a high levodopa dose (1,200 mg/day).

On the last treatment day, animals were sacrificed 18.5 ± 0.68 min after they had started drinking the PD medication (no differences in time of sacrifice were observed between groups: D, 18.55 ± 0.69 min; P, 18.68 ± 0.80 min; R, 18.4 ± 0.67 min; VH, 18.28 ± 0.47 min; one-way ANOVA statistics: *F* = 0.4977, *P* = 0.6865). All rats received their dose supplemented with 1.2% (wt/vol) carmine red to determine their small intestinal motility, and rats in the D and VH groups received, instead of their original dose, on average 7.5/1.875 mg/kg levodopa-carbidopa in order to determine the potential levodopa uptake differences between treated groups. The rats were anesthetized (by isoflurane inhalation anesthesia), and a blood sample was taken (by heart puncture), on average 18.5 ± 0.68 min after the rats had start drinking continuously from the burette (2 to 3 min of drinking). No differences in time of heart puncture were observed between groups (D, 18.55 ± 0.69 min; P, 18.68 ± 0.80 min; R, 18.4 ± 0.67 min; VH, 18.28 ± 0.47 min; one-way ANOVA statistics: *F* = 0.4977, *P* = 0.6865). Blood withdrawn by heart puncture was dispensed in tubes precoated with EDTA at a final concentration of 5 mM and stored on ice during the experiment. The collected blood samples were centrifuged at 1,500 × *g* for 10 min at 4°C, and the plasma was stored at −80°C prior to catecholamine extraction.

The rats were sacrificed by decapitation (using a rodent guillotine), and the small intestine—from the stomach to the cecum—was removed from the abdominal cavity and subsequently dissected, with the first 5 cm representing the duodenum. The remaining part of the small intestine was then dissected into 6 equal sections. The first 3 sections represented the proximal small intestine and the last 3 the ileum. Luminal content was collected by moderate pressing and stored on ice thereafter. Luminal content samples were used for carmine red determination and CFU counting, as described below. Following processing, the samples were snap-frozen in liquid N_2_ and stored at −80°C.

### Carmine red assay.

Part of the luminal content of each small intestinal section was suspended in dimethyl sulfoxide (DMSO; 20% [wt/vol]) and vortexed vigorously. Eight microliters was distributed in a 96-well plate, and the spectrum was measured from 450 to 800 nm (10 nm/step) (carmine red has peaks at 530 and 570 nm). Because of high background differences, the spectrum was linearized between 510 and 590 nm using a fitted line [*y* = *a* × (*x* + *b*)]. The slope (*a*) and the intercept (*b*) were calculated using the data points from 510 and 590 nm, and the calculated value (*x*) for 570 nm (*y*) was subtracted from the measured value. Next, because the animals were not fasting before the treatment, the linearized values were scored in binary fashion; a score of 1 was given when the value was larger than the threshold of 0.003. Finally, the geometric center, concluded to be the most sensitive and reliable measure of intestinal transit (55), was calculated by multiplying the binary score by the segment number (1 to 7, from the end of the stomach to the beginning of the cecum).

### CFU assay.

Contents from the proximal small intestinal segments and ileal segments were mixed and suspended in GM17–17% glycerol medium to preserve bacterial viability after storage at −80°C. The suspended proximal small intestinal and ileal contents were 10-fold serially diluted in phosphate-buffered saline (PBS), and 10 μL was spotted in triplicate on chopped-meat-medium plates (CMM; beef extract, 10 g/L; Casitone, 30 g/L; yeast extract, 5 g/L; K_2_HPO_4_, 5 g/L, menadione, 1 μg/mL, cysteine, 0.5 g/L; hemin, 5 μg/mL, 15 g/L agar), which were incubated for 48 h aerobically and anaerobically (1.5% H_2_, 5% CO_2_, balance with N_2_) in a Coy Laboratory anaerobic chamber (neo-Lab Migge GmbH, Heidelberg, Germany) at 37°C before CFU were counted.

### Catecholamine extraction.

Plasma samples were thawed on ice, and a spatula tip (∼5 mg) of activated alumina powder (199966; Sigma) was added to each well of a 96-well AcroPrep filter plate with 0.2 μM water-wettable polytetrafluoroethylene (wwPTFE) membrane (514-1096; VWR). A 100-μL portion of plasma sample, 1 μM DHBA (3,4-dihydroxybenzylamine hydrobromide) (858781; Sigma) as an internal standard, and 800 μL of TE buffer (2.5% EDTA, 1.5 M Tris-HCl [pH 8.6]) were added sequentially to the wells. Liquid was removed using a 96-well plate vacuum manifold, and the alumina was washed twice with 800 μL of H_2_O. Catechols were eluted using 0.7% HClO_4_, which was incubated for 30 min at room temperature (RT). Samples were injected in an HPLC-ED system (Ultimate 3000 SD high-performance liquid chromatography [HPLC] system coupled to an Ultimate 3000 ECD-3000RS electrochemical detector with a glassy carbon working electrode [DC amperometry at 800 mV]; Thermo Scientific). Samples were analyzed on a C_18_ column (Kinetex; 5 μM, C_18_ 100 Å, 250 by 4.6 mm; Phenomenex, Utrecht, The Netherlands) using a gradient of water-methanol with 0.1% formic acid (0 to 3 min, 99% H_2_O; 3 to 7 min, 99 to 30% H_2_O; 7 to 10 min 30 to 5% H_2_O; 10 to 11 min, 5% H_2_O; 11 to 18 min, 99% H_2_O). A 2-fold serial diluted standard curve ranging from 5 – 0.005 μM (*R*^2^ > 0.97) was used to quantify levodopa (retention time, 4.8 min) and carbidopa (retention time, 8.2 min). Data recording and analysis were performed using Chromeleon software (version 6.8 SR13). Potential intake differences of levodopa were corrected by using carbidopa as an internal standard.

### Levodopa decarboxylation activity test.

Samples stored at −80°C in GM17–17% glycerol were thawed on ice, and 300 μL of 10% (wt/vol) proximal small intestinal or ileal suspensions was washed once with 1 mL of ice-cold PBS to remove levodopa (given during the treatment) and glycerol from the storage medium. Pellets were resuspended in 600 μL enriched beef broth (as described before [50]) supplemented with 20 μg/mL kanamycin (EBB/K), resulting in a 5% (wt/vol) suspension. A 100 μM concentration of levodopa was added to the suspensions, and samples were incubated anaerobically (1.5% H_2_, 5% CO_2_; balance, N_2_) in a Coy Laboratory anaerobic chamber (neo-Lab Migge GmbH, Heidelberg, Germany) at 37°C. Samples of 100 μL were taken at 0 and 24 h and 400 μL of methanol was added. Cells and protein precipitates were removed by centrifugation at 20,000 × *g* for 10 min at 4°C. Supernatant was transferred to a new tube, and the methanol fraction was evaporated in a Savant speed-vacuum dryer (SPD131, Fisher Scientific, Landsmeer, The Netherlands) at 60°C for 90 min. The aqueous fraction was reconstituted to 0.5 mL with 0.7% HClO_4_. Samples were filtered, injected into the HPLC system, and analyzed as described above. A 2-fold serially diluted standard curve ranging from 100 to 1.5625 μM (*R*^2^ > 0.97) was used to quantify levodopa (retention time, 4.8 min) and dopamine (retention time, 4.5 min). Dopamine and levodopa concentrations were quantified from the 24-h samples, and the ratio of dopamine to levodopa was calculated to determine levodopa decarboxylation activity. No dopamine was detected in the baseline (0-h) samples.

### DNA isolation and sequencing.

DNA isolation was performed based on the repeated beat beating (RBB) protocol described in references 65 and 66. Approximately 150 to 200 mg of proximal small intestinal or ileal content was weighted in screw-cap tubes containing ∼0.5 g 0.1-mm glass/silica beads and 3 large 3-mm glass beads. Bacterial cells were lysed by adding 750 μL lysis buffer (NaCl, 500 mM; Tris-HCl [pH 8], 50 mM; EDTA, 50 mM; SDS, 4% [wt/vol]) with sequential bead beating three times for 1 min each time with 1-min intervals on ice in a mini-bead-beater (Biospec, Bartlesville, OK, USA). Samples were incubated for 15 min with regular mixing at 95°C, placed on ice for 5 min, and centrifuged at 20,000 × *g* for 30 min at 4°C. Approximately 600 μL of the samples was recovered and centrifuged again for 5 min. Then, 550 μL was transferred to a new tube containing 200 μL 10 M ammonium acetate and mixed. Samples were incubated on for 5 min ice before centrifugation at 20,000 × *g* for 30 min at 4°C. Approximately 700 μL was transferred to a new tube and centrifuged again for 5 min. Next, 650 μL of supernatant was transferred to a new tube containing 650 μL 2-propanol and mixed. Samples were incubated on ice for 30 min and centrifuged at 20,000 × *g* for 15 min at 4°C. Pellets containing the DNA were washed twice with 800 and 500 μL 70% (vol/vol) ethanol by centrifugation at 20,000 × *g* for 10 min at 4°C. The supernatant was discarded, and the pellet was air dried in a 37°C heat block for 30 min. After drying, the pellets were dissolved in 200 μL TE buffer (1 mM, EDTA, 10 mM Tris-HCl [pH 8]) by vortexing and incubating at 65°C for 10 min. DNA extracted samples were stored at −80°C before further cleanup with the Genomic DNA Clean & Concentrator (gDCC) kit (D4011; Zymo Research, BaseClear Lab Products, The Netherlands). Samples were thawed at RT, and 0.1 mg/mL RNase A (EN0531; Thermo Scientific) was added and incubated for 15 min at 37°C before cleanup with the gDCC kit. Next, chromatin immunoprecipitation (ChIP) binding buffer was added to the RNase A-treated samples (2:1) and mixed, and the mixture was transferred to the gDCC column and subsequently centrifuged at 14,000 × *g* for 30 s at RT. The DNA-bound column was washed twice at 14,000 × *g* for 60 s at RT with wash buffer before being eluted in preheated (65°C) elution buffer which was incubated for 3 min on the column. DNA integrity was checked on agarose gel before samples were sent for 16S (regions V3 and V4) amplicon metagenomic sequencing by Novogene Co., Ltd.

The 16S rRNA gene regions V3 and V4 were amplified with primers 314F (5′-CCTAYGGGRBGCASCAG-3′) and 806R (5′-GGACTACNNGGGTATCTAAT-3′) and with Phusion high-fidelity PCR master mix (New England Biolabs). Amplified products were verified using an Agilent 5400 fragment analyzer, and all passed quality control. PCR products were equally mixed and purified with Qiagen gel extraction kit before libraries for paired-end 250-bp Illumina sequencing were prepared with a NEBNext Ultra DNA library prep kit (New England Biolabs).

### Data analysis.

Paired-end reads were assigned to their samples, and the barcodes and primer sequence were truncated before merging using FLASH (V1.2.7) (67). Quality filtering was performed as described previously (68) using QIIME (v1.7.0) (69). Chimera sequences were removed using the UCHIME algorithm (with the Gold database) (70). Finally, OTU calling was performed using UPARSE (v7.0.1001) (71), and sequences with ≥97% similarity were assigned to the same OTUs. mothur software (72) was used for species annotation at each taxonomic rank (threshold, 0.8 to 1) against the SILVA database (73), and the phylogenetic tree was constructed using MUSCLE (version 3.8.31) (74).

The OTU table and phylogenetic tree were imported in the R package phyloseq (v1.32.0) (75). Richness and diversity were estimated on the raw OTU counts table using phyloseq. For further data analysis, the OTU counts were normalized using the cumulative-sum scaling normalization (CSS) method using the R package metagenomeSeq (v 1.30.0) (76), and taxa were agglomerated at the genus level using phyloseq. Unweighted UniFrac (77) distances were calculated in phyloseq using the phylogenic tree rooted on the longest branch using the root function from R package ape (v5.4-1) (78).

### Statistical analyses.

Data and statistical analyses were performed in GraphPad Prism (v7.0), IBM SPSS Statistics (v 26) or R (v4.0.4) in RStudio (v 1.2.5042). One-way ANOVAs followed by Fisher’s least-significant-difference (LSD) test with FDR correction in Fig. 1 and Table 1 were performed in GraphPad Prism. For the CFU data outliers were determined with the ROUT method (*Q* = 0.1%) and removed using GraphPad Prism. The one-way ANOVA followed by Dunnett’s test in Fig. 2D and E was performed in SPSS. The distance-based redundancy analysis (dbRDA) was performed in phyloseq through the capscale function from the R package vegan (v2.5-6) (79). Significant contributions of the constraints were tested using the anova.cca function in vegan, and environmental vector fitting was performed using the envfit function in vegan. For differential abundance analysis, LDA effect size (LEfSe) (57) analysis was performed in the Galaxy web application (http://huttenhower.sph.harvard.edu/galaxy/). Specific tests and significance are indicated in the figure legends.

### Data availability.

All data generated or analyzed during this study are included in this article and its supplemental material. The 16S rRNA gene metagenomic sequence data were deposited in BioProject under number PRJNA725395.
